# Sequencing at the syllabic and supra-syllabic levels during speech perception: an fMRI study

**DOI:** 10.3389/fnhum.2014.00492

**Published:** 2014-07-08

**Authors:** Isabelle Deschamps, Pascale Tremblay

**Affiliations:** ^1^Département de Réadaptation, Université LavalQuébec City, QC, Canada; ^2^Centre de recherche de l'Institut universitaire en santé mentale de QuébecQuébec City, QC, Canada

**Keywords:** syllabic information, supra-syllabic information, supratemporal plane, speech processing, language

## Abstract

The processing of fluent speech involves complex computational steps that begin with the segmentation of the continuous flow of speech sounds into syllables and words. One question that naturally arises pertains to the type of syllabic information that speech processes act upon. Here, we used functional magnetic resonance imaging to profile regions, using a combination of whole-brain and exploratory anatomical region-of-interest (ROI) approaches, that were sensitive to syllabic information during speech perception by parametrically manipulating syllabic complexity along two dimensions: (1) individual syllable complexity, and (2) sequence complexity (supra-syllabic). We manipulated the complexity of the syllable by using the simplest syllable template—a consonant and vowel (CV)-and inserting an additional consonant to create a complex onset (CCV). The supra-syllabic complexity was manipulated by creating sequences composed of the same syllable repeated six times (e.g., /pa-pa-pa-pa-pa-pa/) and sequences of three different syllables each repeated twice (e.g., /pa-ta-ka-pa-ta-ka/). This parametrical design allowed us to identify brain regions sensitive to (1) syllabic complexity independent of supra-syllabic complexity, (2) supra-syllabic complexity independent of syllabic complexity and, (3) both syllabic and supra-syllabic complexity. High-resolution scans were acquired for 15 healthy adults. An exploratory anatomical ROI analysis of the supratemporal plane (STP) identified bilateral regions within the anterior two-third of the planum temporale, the primary auditory cortices as well as the anterior two-third of the superior temporal gyrus that showed different patterns of sensitivity to syllabic and supra-syllabic information. These findings demonstrate that during passive listening of syllable sequences, sublexical information is processed automatically, and sensitivity to syllabic and supra-syllabic information is localized almost exclusively within the STP.

## Introduction

The speech signal is undoubtedly one of the most complex auditory signals that humans are exposed to, requiring multiple computational steps to parse and convert acoustic waves into discrete linguistic units from which meaning can be extracted. Unsurprisingly, given such complexity, the manner in which the human brain accomplishes the complex computational steps leading to the comprehension of speech remains far from understood.

Functional neuroimaging studies of speech perception offer converging evidence suggesting that the supratemporal plane (STP), and superior temporal sulcus (STS) play a critical role in the processing of speech sounds. This finding is quite robust having been observed under different types of speech perception tasks (i.e., passive listening, monitoring and discrimination tasks as well as neural adaptation paradigms) and with different types of speech stimuli (words, pseudo-words, syllables, phonemes). For instance, neuroimaging studies contrasting the neural activity evoked by speech stimuli to the neural activity associated with the processing of acoustically complex non-speech sounds or silence have reliably reported clusters of activation within the STP and/or STS (Zatorre et al., 1992; Binder et al., 1996, 1997; Dhankhar et al., 1997; Celsis et al., 1999; Burton et al., 2000; Scott et al., 2000; Benson et al., 2001; Vouloumanos et al., 2001; Joanisse and Gati, 2003; Wilson et al., 2004; Liebenthal et al., 2005; Rimol et al., 2005; Wilson and Iacoboni, 2006; Obleser et al., 2007; Okada et al., 2010; Zhang et al., 2011; Tremblay et al., 2012). In addition, neuropsychological evidence demonstrate that bilateral lesions to the superior temporal lobes can result in pure word deafness, a deficit associated with impaired word comprehension but relatively intact ability to process non-speech sounds (Buchman et al., 1986; Tanaka et al., 1987; Poeppel, 1996). While both functional and neuropsychological studies provide strong evidence regarding the importance of the STP and STS for the perception of speech sounds, the specific contribution of each of the sub-regions that form this large cortical area to speech perception is still uncertain; whether it is related to the processing of acoustical, sublexical, or lexical information.

Several neuroimaging studies have contrasted the neural activity evoked by different sublexical units (e.g., consonant clusters, phonemes, syllables) to the processing of non-speech or unintelligible speech sounds (e.g., sinewave analogs, tones, environmental sounds, noise, spectrally rotated syllables, silence) to isolate speech specific processes. These studies reported reliable activation within supratemporal regions [e.g., the superior temporal gyrus (STG), the transverse temporal gyrus (TTG), and planum temporale (PT)], the STS, the middle temporal gyrus (MTG) and, in some instances, in the inferior parietal lobule (IPL), and the inferior frontal gyrus (IFG) (Demonet et al., 1992; Zatorre et al., 1992; Binder et al., 1994; Dhankhar et al., 1997; Giraud and Price, 2001; Vouloumanos et al., 2001; Liebenthal et al., 2005; Rimol et al., 2005; Pulvermuller et al., 2006; Obleser et al., 2007; Tremblay et al., 2012). The consistency of the STP and STS results in studies using words or sublexical units suggest that these regions might be involved in the conversion of acoustical information into phonological representations. However, because these studies have contrasted different types of sublexical units to non-speech or unintelligible speech sounds, the level of processing (e.g., acoustical/phonetic, phonemic, syllabic, supra-syllabic) at which mechanisms implemented within the STP and STS operate remains unclear.

Neuroimaging studies in which phonological mechanisms are engaged by the use of an explicit task (discrimination, rhyming) can more readily target specific mechanisms operating at different sublexical levels (phonemic, syllabic, supra-syllabic) and offer valuable insights into the functional contribution of STP regions to the perception of speech sounds. For instance, STP and STS activation have been reported in studies using a variety of auditory tasks: phonetic discrimination (Burton et al., 2000), rhyming (Booth et al., 2002), syllable identification (Liebenthal et al., 2013), monitoring (Rimol et al., 2005), and phonemic judgments (Jacquemot et al., 2003). Other studies using a neural adaptation paradigm to target phonetic processing have also identified regions within the STP that responded more strongly to stimuli that were part of different phonemic categories than those that felt within the same phonemic category (Dehaene-Lambertz et al., 2005; Joanisse et al., 2007). Taken together, these studies support the notion of a key involvement of the STP and STS in processing sounds at different levels (phonemic, syllabic). However, despite their importance, studies using explicit speech perception tasks requiring judgments on speech sounds probably recruit to greater extent phonological processes than do more naturalistic speech tasks. It is therefore unclear whether similar regions would be recruited in the absence of a task. It is also unclear whether phonological mechanisms operating at different levels (phonemic, syllabic, supra-syllabic) engage the same or different neural networks. Despite the scarcity of studies addressing this issue, in a recent functional magnetic resonance imaging (fMRI) study, McGettigan et al. (2011) manipulated both the complexity of syllabic and supra-syllabic information in pseudo-words during a passive listening task. Syllabic complexity was manipulated by varying the number of consonant clusters (0 vs. 2) and supra-syllabic complexity was manipulated by varying the number of syllables (2 vs. 4). An effect of supra-syllabic complexity was observed in the bilateral PT. However, no positive^1^ effect of syllabic complexity was reported. In contrast, Tremblay and Small (2011), also using fMRI, varied syllabic complexity as indexed by the presence or absence of consonant clusters during the passive listening of words and found that the right PT was sensitive to the syllabic complexity manipulation, supporting the idea that the supratemporal cortex plays a role in processing syllabic information (Grabski et al., 2013).

One question that arises from this literature is whether specific sublexical processes can be localized to specific regions within the STP and STS. In the current experiment, we were interested in investigating the distinct and shared effects of syllabic and supra-syllabic complexity on brain activity during the processing of auditory sequences. To this aim, we parametrically manipulated phonological complexity along two dimensions (1) individual syllable complexity (presence or absence of a consonant cluster in the syllable onset) and (2) sequence-level complexity (the ordering of syllables within a sequence). Given the importance of the STP and STS in the processing of auditory information, we conducted an exploratory anatomical ROI analysis focusing on a fine-grain parcellation of the supratemporal cortex and STS based on our previous work (Tremblay et al., 2012, 2013) to determine whether sub-regions within the STP and STS process similar or different kind of sublexical information during passive speech perception (i.e., syllabic or supra-syllabic). In these prior studies, we demonstrated that sub-regions within the STP exhibited different patterns of sensitivity to speech sounds during speech perception and production, suggesting that the STP contains a mosaic of functionally distinct areas. It is therefore possible that sub-regions within the STP are processing the speech signal in different manners and at different levels, with some focusing on spectral information, while others on syllable- or sequence-level information. Based on the results from our previous studies, we hypothesized that some sub-regions within the STP (in particular the PT) and STS would show similar patterns of activation for both manipulations while others would show a preference for one manipulation. For example, we expected the primary auditory cortex to be sensitive to both manipulations, as both syllabic and supra-syllabic complexity increase acoustic complexity. We also expected the PT to be sensitive to the syllabic manipulation based on previous results (Tremblay and Small, 2011).

## Materials and methods

### Participants

The participants were 15 healthy right-handed (Oldfield, 1971) native French speakers (9 females; 26.8 ± 4.8 years; range 21–34, education 17.3 ± 1.9 years), with normal hearing and no history of language or neurological/neuropsychological disorders. Hearing was assessed using pure tone audiometry (clinical audiometer, AC40, Interacoustic) for each ear separately for the following frequencies: 0.25, 0.5, 1, 2, 3, 4, 5, 8, 12, and 16 kHZ. Then for each participant, a standard pure tone average (PTA: average of threshold at 0.5, 1, and 2 kHz) was computed for the left (17.13 ± 3.78 dB) and right ear (18.68 ± 3.17 dB), since most of the speech sounds fall within this range (Stach, 2010). All participants were screened for depression (Yesavage et al., 1982) and their cognitive functioning was evaluated using the Montreal Cognitive Assessment scale (MOCA) (Nasreddine et al., 2005). All participants were within normal range on the MOCA (i.e., 26/30 or better) and none of the participants were depressive. The study was approved by the committee on research ethics of the Institut Universitaire en santé mentale de Québec (#280-2012).

### Stimuli and task

The experimental task consisted in listening passively (i.e., without performing a task) to sequences of syllables. To investigate sublexical phonological processing, we used sequences of syllables instead of pseudowords to avoid lexical effects. Prior research has demonstrated that pseudowords, given their close resemblance to words, activate regions involved in lexical access and in some cases they do so to an even greater extent than words (Newman and Twieg, 2001; Burton et al., 2005). Thus, the use of pseudowords renders the dissociation between lexical and sublexical phonological processing extremely difficult. For this reason, we decided to used syllable strings rather than words to alleviate potential lexical effects. The degree of complexity of each sequence was manipulated along two phonological dimensions: syllabic and supra-syllabic complexity. Each factor had two levels (simple or complex), resulting in a 2 × 2 experimental design matrix (See Table 1).

**Table 1:** **Experimental conditions**.

**Code**	**Syllable type**	**Sequence type**	**Examples**
SS	Simple	Simple	/ba-ba-ba-ba-ba-ba/
SC	Simple	Complex	/fo-de-ro-fo-de-ro/
CS	Complex	Simple	/kli-kli-kli-kli-kli-kli/
CC	Complex	Complex	/bri-dre-klou-bri-dre-klou/

Syllabic complexity refers to the presence or absence of a consonant cluster (e.g., /gr/): simple syllables were composed of a single consonant and vowel (CV) and complex syllables were composed of a consonant cluster and a vowel (CCV). Supra-syllabic complexity refers to the number of different syllables in a sequence: simple sequences were composed of the same syllable repeated six times (e.g., /ba-ba-ba-ba-ba-ba/) and complex sequences were composed of three different syllables each repeated twice (e.g., /ba-da-ga-ba-da-ga/). While these two manipulations increase phonological complexity, they target different levels of processing; syllabic (individual unit) and supra-syllabic (sequence of units).

All syllables were created by selecting among five frequent French vowels, which included two front vowels (/i/, /ε/), two back vowels (/o/, /u/), and one central vowel (ə), and 12 frequent French consonants, which included four labial consonants (/b/, /p/, /v/, /f/), four coronal consonants (/d/, /n/, /t/, /l/) and four dorsal consonants (/g/, /ɲ/, /k/, /ʁ/). These vowels and consonants were combined to form 60 simple syllables (CV) and 60 complex syllables (CCV). Each syllable was repeated a total of three times (i.e., in three different sequences). Six-syllable sequences were created by producing sequences of three different syllables twice (/pa-ta-ka-pa-ta-ka), or by repeating one syllable six times (/pa-pa-pa-pa-pa-pa/). A native young adult male French speaker from Quebéc City pronounced all syllable sequences naturally in a sound attenuated booth. Each sequence was recorded five times and the best exemplar was selected to use in the experiment. The syllable sequences were recorded at 44.1 KH using a unidirectional microphone connected to a sound card (Fast Track C-400, M-audio), saved directly to disk using Sound Studio 4.5.4 (Felt Tip Software, NY, USA), and edited offline using Wave Pad Sound Editor 4.53 (NHC Software, Canberra, Australia). Each syllable sequence was edited to have an average duration of 2400 ms. The duration of the syllable sequences was the same across all experimental conditions (i.e., 2400 ms). The root mean square (RMS) intensity was then normalized across all sound files. Individual sequences were not repeated during the course of the experiment.

### Procedure

This experimental paradigm resulted in four conditions of 30 trials each, for a total of 120 trials. Each trial lasted 6.5 s. A resting baseline condition was interleaved with the experimental conditions (60 trials). The randomization of the experimental and baseline conditions was optimized using Optseq2 (http://surfer.nmr.mgh.harvard.edu/optseq/). The four conditions were equally divided into two runs. A passive listening experimental paradigm was used; participants were not required to produce any overt response. All stimuli were presented during the delay in acquisition (see Image acquisition section below) using Presentation Software (Neurobehavioral System, CA, USA) through high-quality MRI-compatible stereo electrostatic earplugs (Nordic Neurolab, Norway), which provide 30 dB of sound attenuation.

### Image acquisition

A 3 T Philips Achieva TX MRI scanner was used to acquire anatomical and functional data for each participant. Structural MR images were acquired with a T1-weighted MPRAGE sequence (*TR*/*TE* = 8.2/3.7 ms, flip angle = 8°, isotropic voxel size = 1 mm^3^, 256 × 256 matrix, 180 slices/volume, no gap). Single-shot EPI BOLD functional images were acquired using parallel imaging, with a SENSE reduction factor of 2 to reduce the number of phase encoding steps and speed up acquisition. In order to ensure that syllables were intelligible, a sparse image acquisition technique (Eden et al., 1999; Edmister et al., 1999; Hall et al., 1999; Gracco et al., 2005) was used. A silent period of 4360 ms was interleaved between each volume acquisition. The syllable sequences were presented 360 ms after the onset of the silent period. One hundred and eighty functional volumes were acquired across 2 runs (*TR*/*TE* = 6500/30 ms, volume acquisition = 2140 ms; delay in *TR* 4360 ms, 40 axial slices parallel to AC/PC, voxel size = 3 × 3 × 3, no gap; matrix = 80 × 80; FoV = 240 × 240 mm). This study was part of a larger project, which also included a speech production task and a speech perception in noise task^2^. Those two tasks will not be discussed as part of this manuscript. The speech perception task that is the focus of the present manuscript was always presented first to participants, followed by the speech production task and the speech perception in noise task. Participants were not told until the production task that they would be required to produce speech. This was done in order to avoid priming subvocal rehearsal during the speech perception task. The speech perception in noise task has been reported elsewhere (Bilodeau-Mercure et al., 2014).

### Data analysis

#### fMRI time-series analyses

All functional time-series were motion-corrected, time-shifted, de-spiked and mean-normalized using AFNI (version 10.7, intel 64; Cox, 1996). All time points that occurred during excessive motion (i.e., >1 mm) (Johnstone et al., 2006) were censored. The anatomical scan of each participant was aligned to their registered EPI time series using local Pearson correlations (Saad et al., 2009). The alignment was verified and manually adjusted when necessary. For each participant and for each run a finite impulse response ordinary least squares model was used to fit each time point of the hemodynamic response function for each of the four experimental conditions using AFNI's tent basis function (SS, SC, CS, CC). Additional regressors for the mean, the linear and quadratic trend components as well as the six motion parameters were also included. This model-free deconvolution method allows the shape of the hemodynamic response to vary for each condition rather than assuming a single response profile for all conditions (Meltzer et al., 2008). The interval modeled covered the entire volume acquisition (2.14 s), starting with stimulus onset and continuing at intervals of 6.5 s (i.e., silent period and volume acquisition) for 13 s (i.e., 2 *TR*). All analyses (whole-brain and ROIs) focused on the first interval (i.e., the first TR). The resulting time-series were projected onto the 2-dimensional surfaces where all subsequent processing took place.

For each participant, FreeSurfer was used to create a surface representation of the participant's MRI (Dale et al., 1999; Fischl et al., 1999) by inflating each hemisphere of the anatomical volumes to a surface representation and aligning it to a template of average curvature. SUMA was used to import the surface representations into the AFNI 3D space and to project the pre-processed time-series from the 3-dimensional volumes onto the 2-dimensional surfaces. Both the surface representations and the pre-processed time-series were standardized to a common mesh reference system (Saad et al., 2004). The time-series were smoothed on the surface to achieve a target smoothing value of 6 mm using a Gaussian full width half maximum (FWHM) filter. Smoothing on the surface as opposed to the volume ensures that white matter values are not included, and that functional data located in anatomically distant locations on the cortical surface are not averaged across sulci (Argall et al., 2006).

#### Group-level node-wise analyses

Whole-brain group analyses were performed using SUMA on the participants' beta values resulting from the first level analysis (Saad et al., 2004). The group level analyses focused on (1) the effect of passive auditory sequence perception on the Blood oxygenation level dependent (BOLD) signal (2) the effect of syllabic and supra-syllabic complexity on the BOLD signal during auditory sequence perception, (3) the contrast between the effect of syllabic and supra-syllabic complexity, and (4) the conjunction of the syllabic and supra-syllabic complexity effects. To identify regions recruited during the perception of auditory sequences, a node-wise linear regression was conducted (perception >0, one sample *t*-test option in the AFNI 3dttest++ program). To investigate the effect of syllabic and supra-syllabic complexity, a two-way repeated measure ANOVA (rANOVA) was conducted (AFNI's 3dANOVA program) with syllabic complexity (simple, complex) and supra-syllabic complexity (simple, complex) as within-subjects factors. To identify regions that exhibited a stronger response to one of the manipulations (i.e., syllabic or supra-syllabic), we computed, at the individual subject level, the effect of syllabic complexity (complex syllables - simple syllables) and the effect of supra-syllabic complexity (complex sequences - simple sequences). At the group level, the resulting t-maps were submitted to a paired sample *t*-test, to determine whether the two contrasts (i.e., syllable and sequence contrast) differed (AFNI 3dttest++ program). For the conjunction, we computed a map of the joint activation, for each subject, for syllabic and supra-syllabic complexity (syllabic ∩ supra-syllabic). Only voxels that were significant at *p* = 0.05 (uncorrected) in both individual maps were included in the conjunction map. A group-level average of the conjunction maps was then generated. All resulting group maps were corrected for multiple comparisons using the Monte Carlo procedure implemented in FreeSurfer. This correction implements a cluster-size threshold procedure to protect against Type I error. For the first three analysis, based on the simulation results, it was determined that a family-wise error (FWE) rate of *p* < 0.001 is achieved with a minimum cluster size of 157 contiguous surface nodes, each significant at *p* < 0.01. For the conjunction analysis, we adopted a more lenient correction (a FWE rate of *p* 0.05 was achieved with a minimum cluster size of 202 contiguous surface nodes, each significant at *p* < 0.05).

#### Exploratory anatomical ROI analysis

To examine the role of supratemporal regions in the processing of syllabic and supra-syllabic information, we conducted an exploratory anatomical ROI analysis focusing on a set of 16 a priori selected anatomical regions. In a previous study, using a similar fine-grain parcellation, we demonstrated that several STP regions exhibited differential sensitivity pattern to auditory categories (i.e., syllables or bird songs) and sequence regularity (Tremblay et al., 2012). Here we used a similar parcellation scheme with the addition of the STS to investigate the sensitivity of these regions to syllabic and supra-syllabic information. These bilateral ROIs included the planum polare (PP), the STG, the STS, the TTG, the transverse temporal sulcus (TTS), the PT, the caudal segment of the Sylvia fissure (SF). These ROIs were anatomically defined on the participant's individual cortical surface representation using an automated parcellation scheme (Fischl et al., 2004; Desikan et al., 2006). This parcellation scheme relies on a probabilistic labeling algorithm based on the well-established anatomical convention of Duvernoy (1991). The anatomical accuracy of this method is high, approaching that of manual parcellation (Fischl et al., 2002, 2004; Desikan et al., 2006). The advantage of using anatomical (as opposed to functional) ROIs based on individual micro-anatomical landmarks is that it can capture inter-subject anatomical variability, something that is loss when using normalized templates (i.e., functional ROIs based on group level data or cytoarchitectonic maps). It is also more anatomically precise. Thus, given that we were specifically interested in exploring the functional anatomy of the STP/STS, the choice of an anatomical ROI approach was logical.

**Figure 1:**
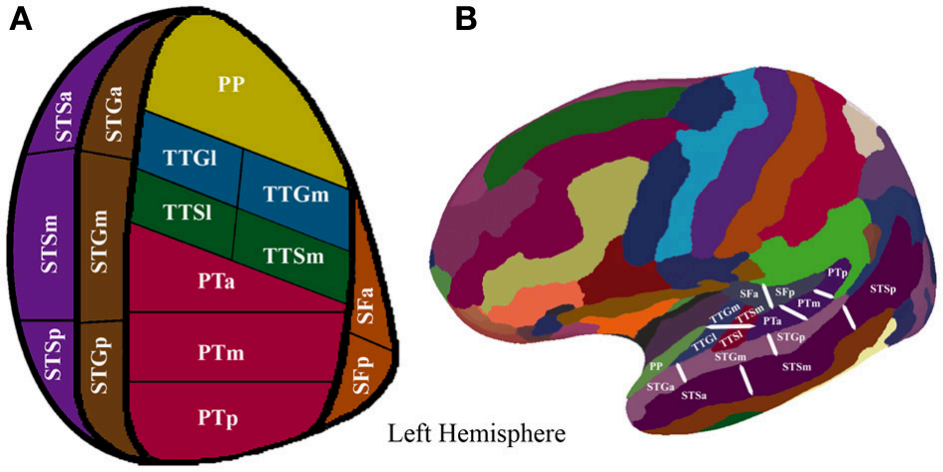
**(A)** Supratemporal and STS parcellation. Anatomical parcellation of the STP and STS displayed on a flattened schematic representation of the supratemporal cortex surface. **(B)** Anatomical parcellation of the supratemporal plane displayed on a lateral view of a left hemisphere smoothed white matter inflated surface.

To augment the spatial resolution of the FreeSurfer anatomical parcellation, we manually subdivided the initial parcellation of each participant's inflated surface in the following manner: the STS, the STG, the PT were subdivided into equal thirds whereas the SF, the TTG, and the TTS were subdivided into equal halves, resulting in 16 ROIs (refer to Figure 1 and Table 2 for details). The use of this modified FreeSurfer parcellation scheme is advantageous for several reasons: (1) it is based on a well-recognized anatomical parcellation scheme, (2) it is systematic, (3) it is easily replicable across participants and studies, and (4) it has been shown to reveal functional subdivisions within the STP (Tremblay et al., 2012).

**Table 2:** **Surface description of the ROI parcellation**.

**Regions**	**Description**
Superior temporal sulcus (STS)	The FreeSurfer STS ROI is bounded anteriorly by the temporal pole, medially by the STG, laterally by the MTG, and posteriorly by the IPL. We divided this region into roughly equal thirds along the rostro-caudal axis (STGp, STGm, STGa)
Superior temporal gyrus (STG)	The FreeSurfer STG ROI runs from the rostral edge of the STS to the supramarginal gyrus. It is bounded medially by the SF. We divided this region into roughly equal thirds along the rostro-caudal axis (STGp, STGm, STGp)
Planum temporale (PT)	The FreeSurfer PT ROI is bounded anteriorly by the TTS, medially by the SF, laterally by the STG, and posteriorly by the supramarginal gyrus. We divided this region into roughly equal thirds along the rostro-caudal axis (PTp, PTm, PTa)
Transverse temporal sulcus (TTS)	The FreeSurfer TTS ROI is bounded posteriorly by the PT and anteriorly by the TTG. We divided this region into two halves along the medial-lateral axis
Transverse temporal gyrus (TTG)	The FreeSurfer TTG ROI is bounded rostrally by the rostral extent of the TTS, caudally by the caudal portion of the insular cortex, laterally by the STG and medially by the SF. We divided this region in roughly equal halves along a medial-lateral axis
Caudal segment of the Sylvian Fissure (SF)	The FreeSurfer posterior SF ROI runs from the lower end of the central sulcus to the end of the posterior ascending ramus (Dahl et al., 2006). We divided this region in roughly equal halves
Planum polare (PP)	Unedited version of FreeSurfer. It is bounded rostrally by the temporal pole, caudally by the TTG, and medially by the parahippocampal gyrus

For each participant, we extracted the mean percentage of BOLD signal change in each of the 16 resulting bilateral ROIs. First, we determined which ROIs were significantly active during the auditory perception of the sequences by testing the following hypothesis using FDR-corrected *t*-tests (Benjamini and Hochberg, 1995; Genovese et al., 2002) (*q* = 0.05): (i) perception >0, (*n* = 32, one-sample *t*-tests).

For each ROI that was significantly active, we conducted a three-way ANOVA with repeated measurements on the magnitude of the BOLD signal as a function of hemisphere, syllabic complexity, and supra-syllabic complexity. Within each ROI, all main effects as well as two-way and three-way interactions were examined using Bonferroni corrected paired-sample *t*-tests (α = 0.05). Adjusted *p*-values are reported.

## Results

### Whole brain results

**Figure 2:**
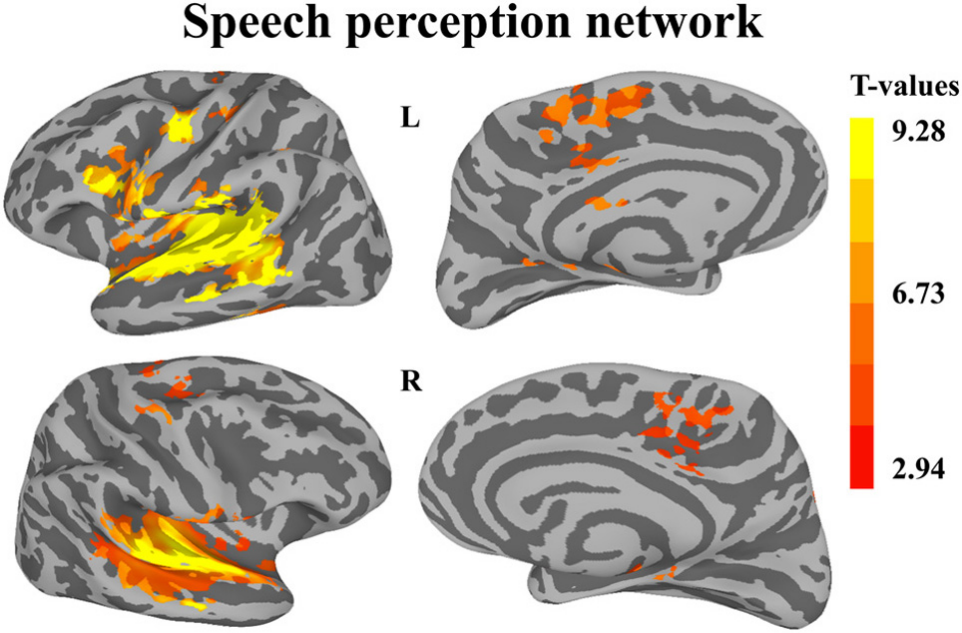
**Whole-brain analysis of BOLD response illustrating regions significantly active during speech perception**. Activation is shown on the group average smoothed flattened surfaces. All analyses are controlled for multiple comparisons using a cluster extent of 157 vertices, and a single node threshold of *p* < 0.01, to achieve a family-wise error rate of *p* < 0.001.

The first whole-brain analysis focused on identifying brain regions that were significantly recruited during the perception of auditory sequences regardless of syllabic and supra-syllabic complexity. The node-wise linear regression identified regions within the bilateral precentral gyrus, IFG, medial superior frontal gyrus and supratemporal cortex, as well as the left cingulate gyrus and right superior frontal gyrus that were more active than during the perception of auditory sequences than the baseline (i.e., rest) (for details, refer to Figure 2 and Table 3).

**Table 3:** **FWE-corrected whole-brain for the speech perception network**.

**Anatomical location**	**Hemi**	***x***	***y***	***z***	***F*-value**	***p*-value**	**Cluster size (nodes)**	**Area (mm)**
**AUDITORY SEQUENCES > REST**								
STGa extending into the MTG, STGp, SMG and circular sulcus of the insula (multiple clusters)	Left	−54	3	−7	9.29	*p* < 0.00001	14,323	4808
Precentral gyrus extending into the central sulcus and the inferior frontal gyrus		−58	2	19	5.91	*p* < 0.00001	2300	855
Medial superior frontal gyrus		−7	−4	56	7.29	*p* < 0.00001	1589	439
Precentral gyrus		−51	−6	45	5.74	0.00004	863	252
Cingulate gyrus and sulcus		−4	−12	38	4.59	0.00035	543	155
Central sulcus		−36	−27	48	4.29	0.00064	442	149
Lateral-occipito-temporal sulcus		−43	−50	−10	5.31	0.00009	397	121
Parieto-occipital sulcus		−15	−58	14	3.84	0.002	191	53
Supramarginal gyrus		−50	−44	47	4.74	0.00026	180	41
Cingulate gyrus		−12	−41	1	3.52	0.0031	158	24
STGm extending into the TTGl, STGa, MTG, and STSp	Right	61	−22	2	11.28	*p* < 0.00001	11,839	4200
Cingulate gyrus and sulcus (multiple clusters)		15	−23	45	6.94	*p* < 0.00001	791	177
Subcentral gyrus and sulcus		58	−4	11	4.67	0.0003	660	171
Medial superior frontal gyrus (multiple clusters)		8	−24	53	4.38	0.0005	524	143
Central sulcus		45	−9	38	7.74	*p* < 0.00001	270	102
Inferior circular sulcus of the insula and PP (two clusters)		42	0	−20	4.62	0.0003	363	100
Middle frontal gyrus and precentral sulcus (two clusters)		41	1	47	5.34	0.00008	359	86
Superior circular sulcus of the insula		38	−16	22	5.29	0.00009	171	61
Central sulcus		31	−28	50	3.87	0.0015	196	51
Superior frontal sulcus		25	0	47	4.33	0.00059	176	50
Precentral sulcus		19	−9	62	6.10	0.00002	204	47
Superior temporal sulcus		48	−54	21	−6.21	0.00002	297	41
Superior parietal gyrus		13	−74	44	4.94	0.0002	205	40
Parahippocampal gyrus		19	−31	−8	4.32	0.0006	201	39

All coordinates are in MNI space and represent the peak surface node for each of the cluster (FWE: p = 0.001, minimum cluster size: 157 contiguous surface nodes, each significant at p < 0.01). When more than one activation foci is listed, this means than the cluster had multiple peaks or was not continuous.

**Figure 3:**
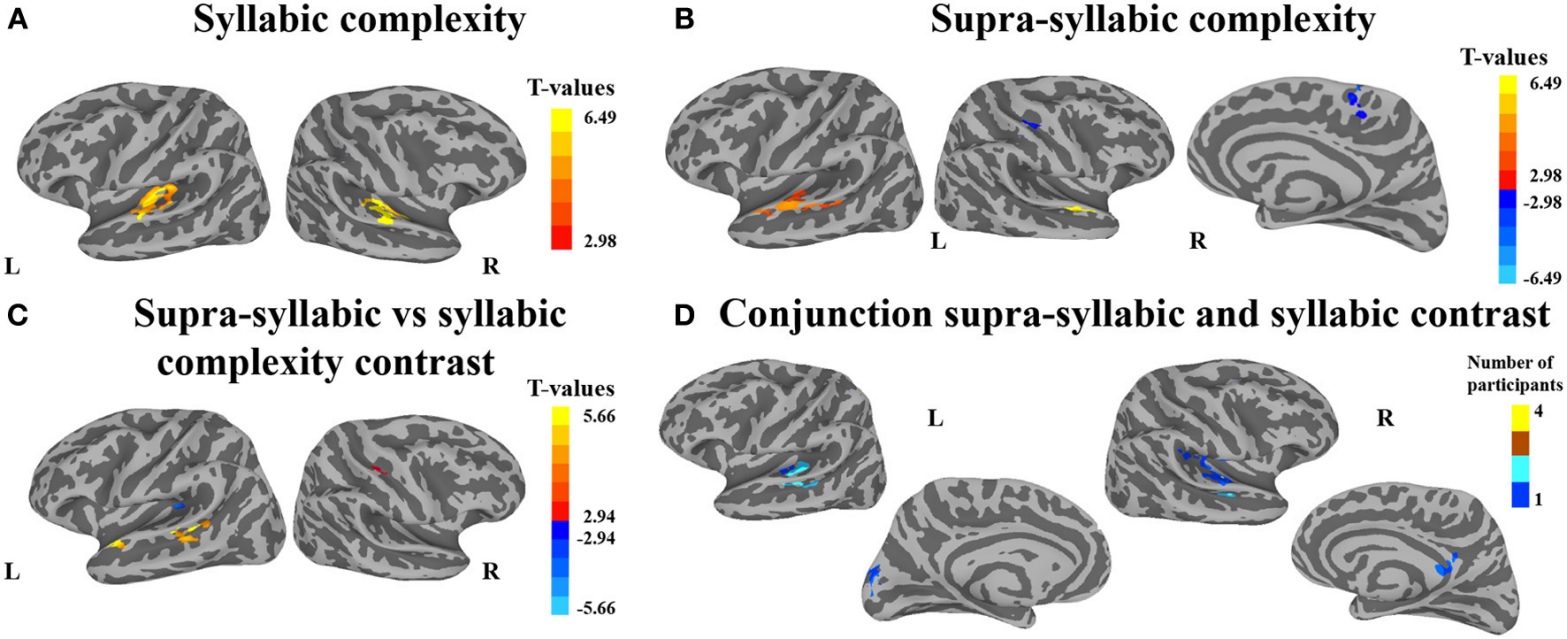
**Whole-brain analysis of BOLD response**. Activation is shown on the group average smoothed flattened surfaces. The first three analyses **(A–C)** are controlled for multiple comparisons using a cluster extent of 157 vertices, and a single node threshold of *p* < 0.01, to achieve a family-wise error rate of *p* < 0.001. The last analysis **(D)** is controlled for multiple comparisons using a cluster extent of 202 vertices, and a single node threshold of *p* < 0.05, to achieve a family-wise error rate of *p* < 0.05. Panel **(A)** illustrates regions significantly active for the contrast between levels of syllabic complexity (complex > simple sequences). Panel **(B)** illustrates regions significantly active for the contrast between levels of supra-syllabic complexity (complex > simple). Panel **(C)** illustrates regions that were differently active for the two complexity contrasts ([complex sequence - simple sequence] - [complex syllable - simple syllable]). Panel **(D)** illustrates regions significantly active for the conjunction of syllabic and supra-syllabic complexity (syllabic complexity ∩ supra-syllabic complexity). The color scheme represents the number of participants in which an overlap between the two manipulations was found (less than 5).

The second analysis sought to identify brain regions that were sensitive to syllabic complexity, supra-syllabic complexity. The node-wise rANOVA showed significant main effects of syllabic complexity and supra-syllabic complexity within the STP (for details, refer to Table 4 and Figures 3A,B). As illustrated in Figure 3A, for the syllabic complexity manipulation, significant clusters of activation were observed within the left TTGl extending posteriorly into the SFp, and medially into the inferior sulcus of the insula as well as the right TTGl extending posteriorly into the SFa, laterally into the STGm and medially into the inferior circular sulcus of the insula (for details, refer to Table 4A). These two regions were significantly more active for the complex syllables than the simple syllables. As illustrated in Figure 3B, an effect of supra-syllabic complexity was found within the left STGm extending medially into the STSm, and TTSl as well as the right STGa/m, the right central sulcus and the right superior frontal gyrus. Only the clusters within the STP were significantly more active for the complex sequences (see Table 4B). No significant two-way interaction between syllabic complexity and supra-syllabic complexity was found.

**Table 4:** **FWE-corrected whole-brain BOLD results**.

**Anatomical location**	**Hemi**	***x***	***y***	***z***	***t*-value**	***p*-value**	**Cluster size (nodes)**	**Area (mm)**
**(A) SYLLABIC COMPLEXITY**								
TTGl extending into the SFp and the inferior circular sulcus of the insula	Left	−55	−14	2	5.84	0.00005	1618	596
TTGl extending into the SFa, STGm, and inferior circular sulcus of the insula	Right	63	−14	4	5.26	0.0001	1106	370
**(B) SUPRA-SYLLABIC COMPLEXITY**								
STGm extending into the TTSl and STSm (multiple clusters)	Left	−60	−12	−5	6.49	0.00002	1277	443
STGm and STGa	Right	61	−4	−4	5.91	0.00004	240	83
Central sulcus		37	−19	42	−4.71	0.0004	266	79
Superior frontal gyrus (multiple clusters)		8	−26	54	−6.82	0.000008	249	65

All coordinates are in MNI space and represent the peak surface node for each of the cluster (FWE: p = 0.001, minimum cluster size: 157 contiguous surface nodes, each significant at p < 0.01). T-values are reported instead of F-values. T-values were obtained by contrasting the two levels of complexity for each experimental factor while collapsing across the other one. When more than one activation foci is listed, this means than the cluster had multiple peaks or was not continuous.

The third analysis sought to determine whether brain regions responded more to one complexity manipulation than the other. The node-wise *t*-test showed that the effect of supra-syllabic complexity was stronger than the effect of syllabic complexity within STP regions in the left STSp, STGp, and STGa, whereas the effect of syllabic complexity was stronger than the effect of supra-syllabic complexity in the left TTGl (for details, refer to Table 5 and Figure 3C).

**Table 5:** **FWE-corrected whole-brain BOLD results**.

**Anatomical location**	**Hemi**	***x***	***y***	***z***	***t*-value**	***p*-value**	**Cluster size (nodes)**	**Area (mm)**
**SUPRA-SYLLABIC COMPLEXITY > SYLLABIC COMPLEXITY**								
STSp and STGp (two clusters)	Left	−50	−44	0	5.66	0.00005	600	196
STGa		−56	−2	−7	4.93	0.0002	172	78
TTGl		−37	−38	15	−4.63	0.0003	172	58
Central sulcus	Right	1	−11	16	−4.25	0.0007	211	60

All coordinates are in MNI space and represent the peak surface node for each of the cluster (FWE: p = 0.001, minimum cluster size: 157 contiguous surface nodes, each significant at p < 0.01). Two clusters or multiple clusters indicate that the activation cluster is not continuous.

The last analysis focused on identifying regions that were sensitive to both experimental manipulations. As illustrated in Figure 3D, the conjunction between the syllabic complexity contrast and the supra-syllabic contrast revealed overlapping activation for both experimental manipulation within left STP regions (TTSm, TTSl, PTa, STGm), the cuneus as well as right STP regions (TTSm, TTSl, SFp), the right supramarginal gyrus, and the right subparietal sulcus. For each area that responded to both manipulations, we quantified the number of participants for which the two effects overlapped. As can be seen in Figure 3D, less than five participants shared common overlapping regions.

### Exploratory supra-temporal ROI analyses

Only the ROIs that were significantly activated for speech perception were included in the subsequent analyses. Of the 32 ROIs, only the bilateral STSp was not significantly activated. For each remaining ROI (*n* = 15), we investigated the main effects of hemisphere, syllabic complexity, supra-syllabic complexity as well as the two-way interactions between hemisphere ^*^ syllabic complexity, hemisphere ^*^ supra-syllabic complexity, syllabic complexity ^*^ supra-syllabic complexity and three-way interaction between hemisphere ^*^ syllabic complexity ^*^ supra-syllabic complexity. Bonferroni adjusted *p*-values are reported.

**Figure 4:**
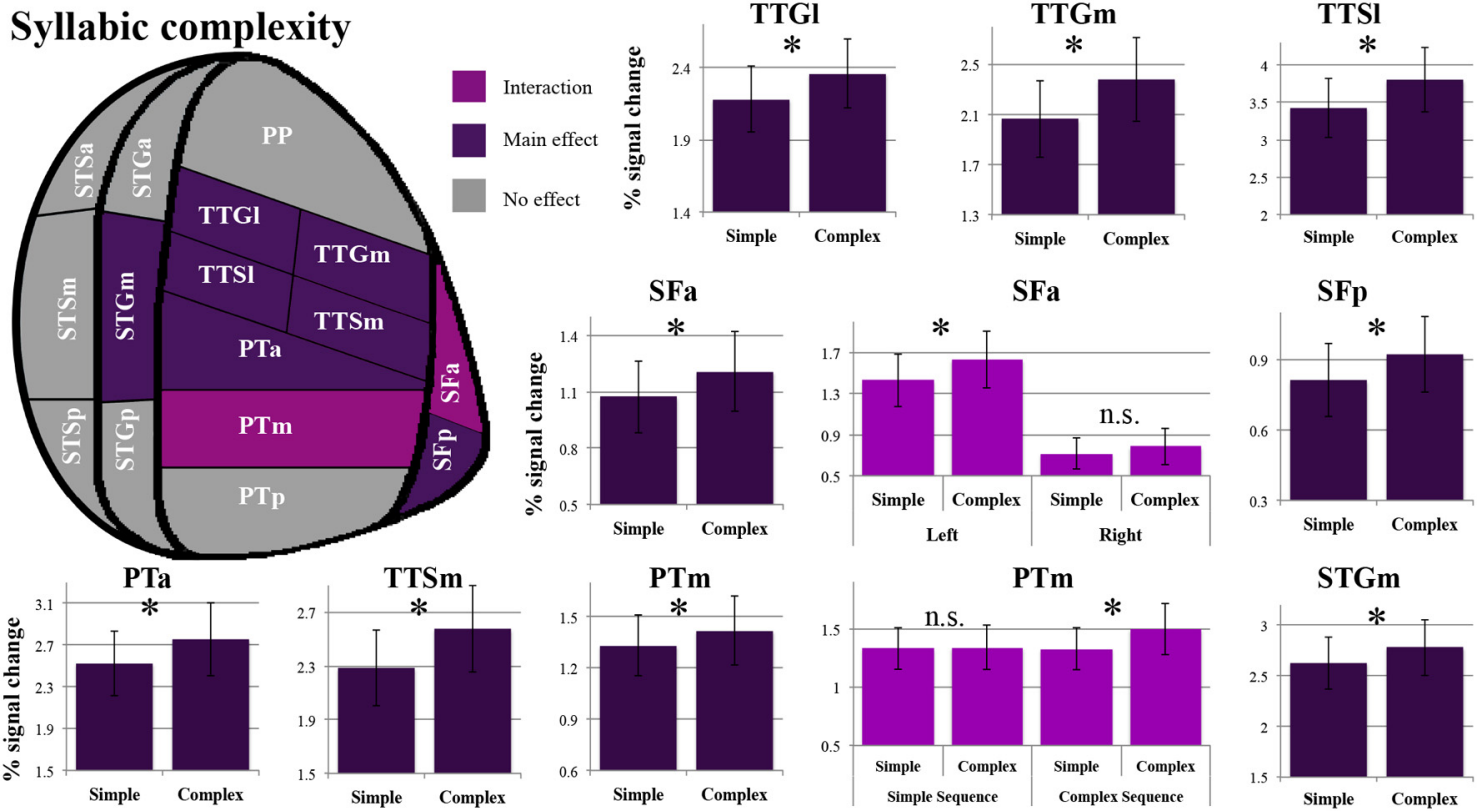
**Patterns of syllabic complexity effects observed in exploratory STP and STS ROI analysis**. The results are shown on a flattened schematic representation of STP and STS showing the parcellation used in this study (different areas shown not to scale). Areas in dark purple exhibited a main effect of complexity and areas in lighter purple exhibited an interaction was observed (hemisphere ^*^ syllabic complexity for the SFa and syllabic complexity ^*^ supra-syllabic complexity for the PTm). Legend: PP, planum polare; TTG, transverse temporal gyrus (*m*, medial; l, lateral); TTS, transverse temporal sulcus (m, medial; l, lateral); PT, planum temporale (a, anterior; m, middle; p, posterior); SF, caudal Sylvian fissure (a, anterior; p, posterior); STG, superior temporal gyrus (a, anterior; m, middle; p, posterior); STS, superior temporal sulcus (a, anterior; m, middle; p, posterior); ^*^significant contrast at p_FWE_ = 0.05, Bonferonni corrected; n.s. non-significant contrast. Error bars represent standard error of the mean.

As shown in Figure 4, a main effect of syllabic complexity was observed in the TTGl [*F*_(1, 14)_ = 26.44, *p* = 0.0002], the TTGm [*F*_(1, 14)_ = 31.11, *p* = 0.00007], the TTSl [*F*_(1, 14)_ = 29.4, *p* = 0.00009], the TTSm [*F*_(1, 14)_ = 17.13, *p* = 0.001], the STGm [*F*_(1, 14)_ = 8.71, *p* = 0.011], the SFp [*F*_(1, 14)_ = 5.90, *p* = 0.029], the SFa [*F*_(1, 14)_ = 9.84, *p* = 0.007], the PTa [*F*_(1, 14)_ = 13.61, *p* = 0.002] and the PTm [*F*_(1, 14)_ = 4.84, *p* = 0.045]. We then determined the type of stimuli driving the effect. For all nine regions, a stronger effect was observed for complex than simple syllables (paired sample *t*-tests, Bonferroni corrected). For the SFa, a significant hemisphere ^*^ syllabic complexity interaction was also observed [*F*_(1, 14)_ = 8.39, *p* = 0.012]. Paired sample *t*-tests revealed that the source of the interaction was due to the presence of an effect of syllabic complexity for the left SFa (*t* = 4.39, *p* = 0.003) but not the right SFa (*t* = 1.358, *p* = 0.59) (for details, refer to Figure 3). For the PTm, a significant syllabic complexity ^*^ supra-syllabic complexity interaction was noted. Paired sample *t*-tests revealed that this interaction was due to the presence of an effect of syllabic complexity for the complex (*t* = 2.95, *p* = 0.044) but not the simple sequences (*t* = 0.01, *p* = 1) (for details, refer to Figure 4). For the TTSm, a significant syllabic complexity ^*^ hemisphere interaction was observed. Paired sample *t*-tests revealed that this interaction was due to a marginally significant difference when we computed a differential complexity score per hemisphere [complex - simple syllable] and compared these scores across hemispheres (*t* = −2.51, *p* = 0.06). A significant three-way interaction was observed in the STSa. To investigate the source of the three-way interaction, two-way interactions were computed. A two-way interaction between syllabic complexity and hemisphere was found for complex sequences [*F*_(1, 14)_ = 7.32, *p* = 0.018] but not for simple sequences [*F*_(1, 14)_ = 0.413, *p* = 0.531]. Paired sample *t*-tests were computed. A marginally significant difference (*t* = −2.67, *p* = 0.054) was found when we computed a differential complexity score per hemisphere [complex - simple syllable] and compared these scores across hemispheres.

The overall pattern that emerges with regard to the syllabic manipulation is a significant increase in sensitivity for complex syllables (i.e., CCV) relative to simple syllables (i.e., CV) in the TTGl, TTGm, TTSm, TTSl, STGm, SFp, SFa, PTa, and PTm. Furthermore, the SFa demonstrated a lateralization effect during the processing of syllabic information (the left SFa was sensitive to the syllabic manipulation but not the right SFa). Lastly, the PTm was the only region where an interaction between the syllabic and supra-syllabic manipulations was observed. In this region, the effect of syllabic complexity was restricted to complex sequences.

**Figure 5:**
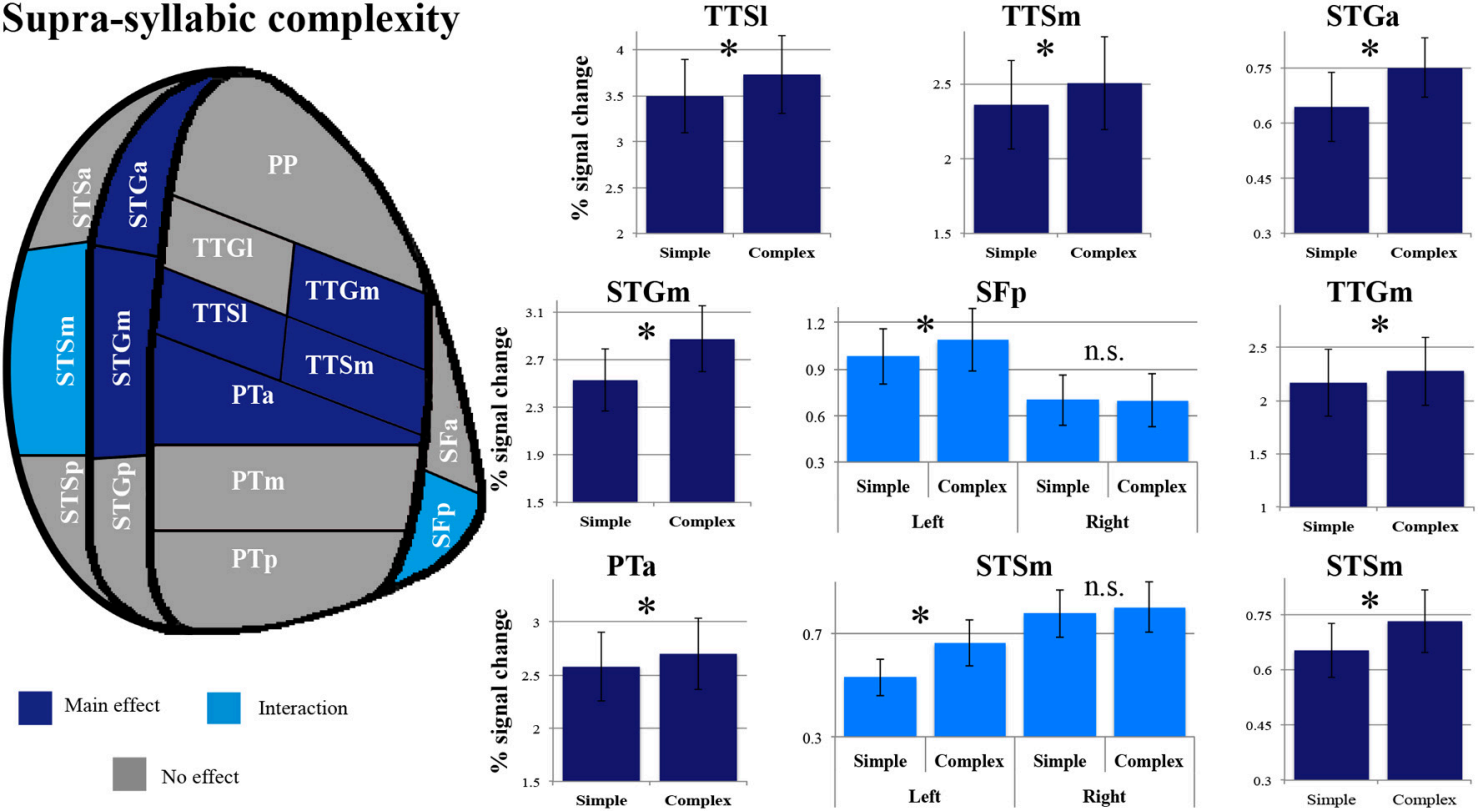
**Patterns of supra-syllabic complexity effects observed in exploratory STP and STS ROI analysis**. The results mapped onto a flattened schematic representation of STP and STS showing the parcellation used in this study (different areas shown not to scale). Areas in dark blue represent a main effect of complexity and areas in lighter blue represent areas where an interaction was observed (hemisphere ^*^ syllabic complexity for the SFp and STSm). Legend: PP, planum polare; TTG, transverse temporal gyrus (m, medial; l, lateral); TTS, transverse temporal sulcus (m, medial, l, lateral); PT, planum temporale (a, anterior; m, middle; p, posterior); SF, caudal Sylvian fissure (a, anterior, p, posterior); STG, superior temporal gyrus (a, anterior, m, middle, p, posterior); STS, superior temporal sulcus (a, anterior, m, middle, p, posterior); ^*^significant contrast at p_FWE_ = 0.05, Bonferonni corrected; n.s. non-significant contrast. Error bars represent standard error from the mean.

As shown in Figure 5, a main effect of supra-syllabic complexity was observed in the STSm [*F*_(1, 14)_ = 5.89, *p* = 0.03], the STGa [*F*_(1, 14)_ = 5.39, *p* = 0.036], the STGm [*F*_(1, 14)_ = 27.38, *p* = 0.0001], the PTa [*F*_(1, 14)_ = 8.64, *p* = 0.01], the TTSl [*F*_(1, 14)_ = 10.95, *p* = 0.005], the TTSm [*F*_(1, 14)_ = 11.67, *p* = 0.004], and the TTGm [*F*_(1, 14)_ = 8.619, *p* = 0.011]. We determined that for all seven regions, the complex sequences were driving the main effect of supra-syllabic complexity as they elicited higher levels of BOLD signal than simple sequences (paired sample *t*-tests, Bonferroni corrected). For the STSm and SFp, a hemisphere ^*^ supra-syllabic interaction was observed [STSm: *F*_(1, 14)_ = 10.06, *p* = 0.007, SFp:*F*_(1, 14)_ = 11.84, *p* = 0.004]. For both regions, paired sample *t*-tests revealed that the source of the interaction was due to an effect of supra-syllabic complexity in the left hemisphere (STSm: *t* = 3.851, *p* = 0.004, SFp: *t* = 2.55, *p* = 0.046) but not the right hemisphere (STSm: *t* = 0.64, *p* = 1, SFp: *t* = 0.965, *p* = 1).

The overall pattern that emerges with regard to the supra-syllabic manipulation is a significant increase in sensitivity for complex sequences (i.e., three different syllables) relative to simple sequences (i.e., same syllable repeated 3×) in the STSm, STGa, STGm, PTa, TTSl, TTSm, and TTGm. In addition, in two regions, the STSm and SFp an effect of hemisphere was observed. For both of these regions, the effect of supra-syllabic complexity was only observed in the left hemisphere.

**Figure 6:**
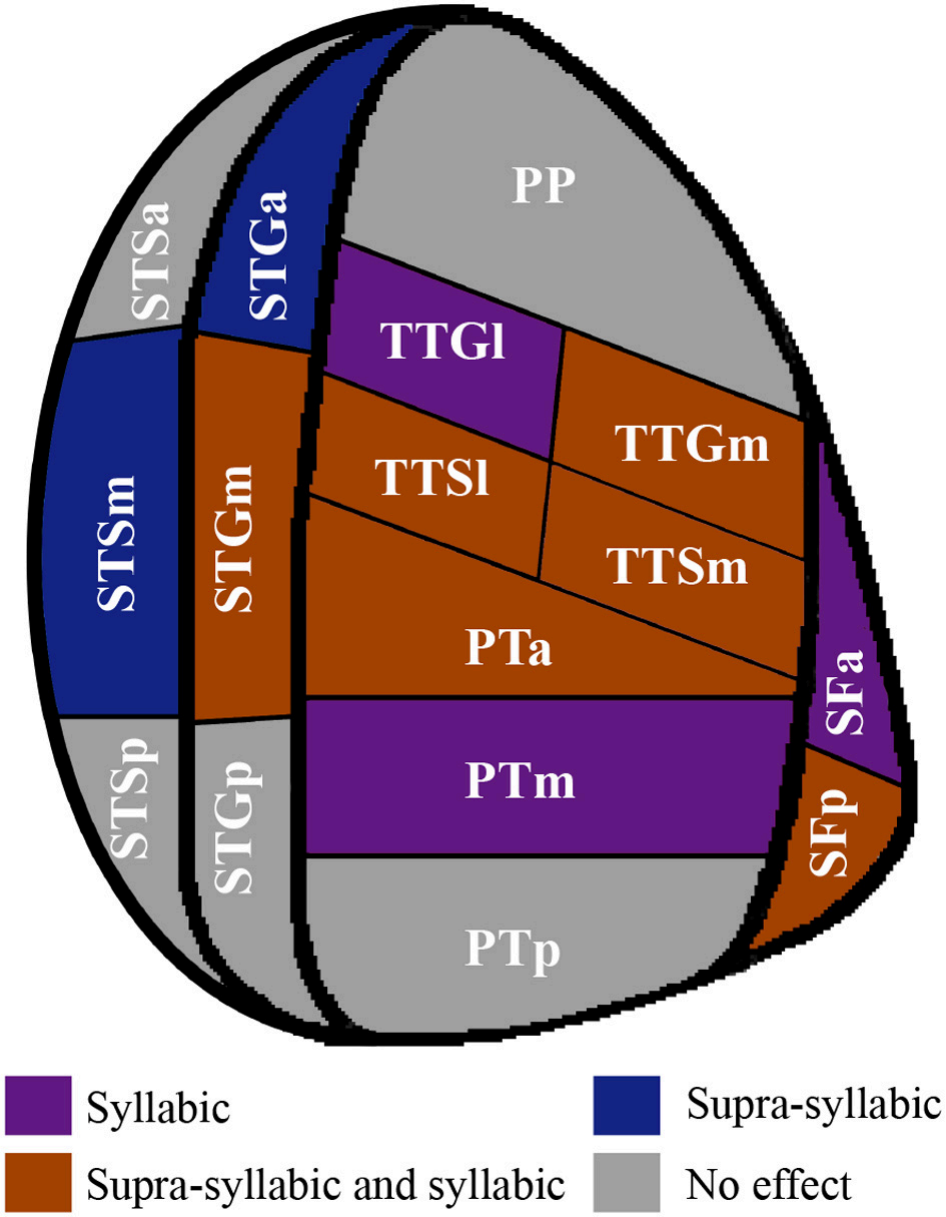
**Patterns of main effects of syllabic and supra-syllabic complexity observed in exploratory STP and STS ROI analysis**. The results are shown on a flattened schematic representation of STP and STS showing the parcellation used in this study (different areas shown not to scale). Areas in dark purple are those that exhibited a main effect of syllabic complexity, areas in blue are those that exhibited a main effect of supra-syllabic complexity, areas in orange are those that exhibited where both an effect of syllabic and supra-syllabic complexity were observed and areas in gray are those that exhibited no effect was observed. Legend: PP, planum polare; TTG, transverse temporal gyrus (m, medial, l, lateral); TTS, transverse temporal sulcus (m, medial, l, lateral); PT, planum temporale (a, anterior, m, middle, p, posterior); SF, caudal Sylvian fissure (a, anterior, p, posterior); STG, superior temporal gyrus (a, anterior, m, middle, p, posterior); STS, superior temporal sulcus (a, anterior, m, middle, p, posterior).

In sum, the pattern that emerges from the ROI analysis suggest that some ROIs (STGm, TTSl, TTGm, TTSm, PTa) are sensitive to both experimental manipulations while others are only sensitive to one experimental manipulation (i.e., syllabic: left SFa, PTm, TTGl; supra-syllabic: left STSm, left SFp; for details refer to Figure 6). In addition, for ROIs that were sensitive to both manipulations, the magnitude of the manipulations was equivalent given the absence of syllabic complexity ^*^ supra-syllabic complexity interaction within these regions.

## Discussion

Neuroimaging studies have consistently documented the role of two large and functionally heterogeneous cortical areas, the STP and STS, in the perception of speech sounds. However, a detailed understanding of the role STP and STS in the processing of sublexical information has not yet emerged. This is largely related to the intrinsic complexity of the speech signal. Indeed, comprehending speech requires the interaction of complex sensory, perceptual, and cognitive mechanisms. The question, then, that naturally arises is whether these regions shows differential patterns of activation as of function of the type of information being processed (syllabic vs. supra-syllabic) (functional heterogeneity) and the specific sub-region (spatial heterogeneity).

The main objective of the current study was to examine, using fMRI, whether the processing of syllabic and supra-syllabic information during a passive listening task involve similar or distinct networks, with an emphasis on the STP and the STS. A passive listening paradigm was used in order to minimize task-related cognitive/executive demands. Given the importance of the STP and STS in speech processing, we conducted an exploratory ROI analysis focusing on 16 bilateral STP/STS sub-regions to determine whether differential patterns of activation would be observed as a function of the type of information processed (i.e., syllabic or supra-syllabic). To preface the discussion, the results from the whole-brain analysis identified a network of regions involved in the perception of speech sounds that is consistent with previous neuroimaging studies that contrasted the processing of sublexical speech units to rest (Benson et al., 2001; Hugdahl et al., 2003; Wilson et al., 2004; Rimol et al., 2005; Wilson and Iacoboni, 2006). In addition, the results clearly demonstrate that the processing of auditory syllable sequences recruits both the left and right hemisphere, consistent with the notion that the processing of speech sounds is bilateral (Hickok and Poeppel, 2004, 2007; Hickok, 2009). The highly consistent results from the whole-brain and ROI analysis demonstrate that both syllabic and supra-syllabic information are processed during passive listening. The anatomical specificity afforded by the ROI analyses allowed us to go further in exploring the specific functional contribution of sub-regions within the STP and STS during the perception of speech sounds. The findings are discussed below.

Results from the whole-brain analyses demonstrate widespread bilateral supratemporal activation resulting from the syllabic manipulation. The widespread extent of this activation was not expected based on previous fMRI results (McGettigan et al., 2011; Tremblay and Small, 2011). Of the few studies that have investigated the effect of consonant clusters during passive speech perception, in one study, activation within the right PT was scaled to syllabic complexity (Tremblay and Small, 2011) and in the other, no positive effect was reported (McGettigan et al., 2011). Our finding of widespread supratemporal effects may be related to the type of stimuli used. While in the present study we used meaningless sequences of syllables, Tremblay and Small (2011) used whole words, for which the mapping of sounds to linguistic representations may be more automatic, requiring less resources for the processing of syllabic information. However, if the processing of syllabic information interacts with lexical status, an effect of complexity should have been observed in the McGettigan et al. (2011) study given that pseudo-words were used, which are not overlearned stimuli with a stored lexical representation. It is possible that the absence of an effect of syllabic complexity in the latter study is attributable to a less salient experimental manipulation. In the present study, we contrasted sequences of syllables with either six or no consonant clusters, yielding a very robust effect. Although the use of a passive listening paradigm minimized attention-directed processes, mimicking more closely naturalistic speech perception situations, the use of syllable as experimental stimuli might have taxed to a greater extent phonological processes than the use of pseudo-words and words. This line of reasoning is consistent with neuropsychological and neurophysiological evidence suggesting that language comprehension does not depend on the processing of sublexical units (i.e., units smaller than words, such as syllables, phonemes, and phonetic features). For instance, it has been shown that patients with good word-level auditory comprehension abilities can fail on syllable and phoneme discrimination tasks (Basso et al., 1977; Boatman et al., 1995). Similarly, electrocortical mapping studies have provided evidence that phonological processes (e.g., syllable discrimination) and auditory word comprehension processes are not entirely circumscribed to the same STP regions (for a review, refer to: Boatman, 2004). In sum, while syllabic complexity effects are observed in sequences of syllables, further research need to determine whether and how syllabic information contributes to the perception of speech sounds and language comprehension.

Both whole-brain and exploratory ROI analyses identified a region that was sensitive to the presence or absence of consonant clusters; the lateral part of the primary auditory cortex (TTGl). In addition, the exploratory ROI analysis also identified the left SFa and PTm, as regions being sensitive to the syllabic manipulation. These results tentatively suggest that this effect stems from the addition of an extra consonant in the onset of the syllable and not from differences between adjacent syllables (i.e., two different syllables). This pattern of response is consistent with the hypothesis that these regions are sensitive to the structure of the syllable (i.e., whether it is phonologically complex or not). Whether these regions respond to the complexity of the syllabic structure in general or to a specific component of the syllable (i.e., onset, rhyme, nucleus, or coda) however remains to be determined. Though the specific contribution of these three regions in the processing of syllabic information is still awaits further specifications, these three regions are nonetheless robustly activated during the perception of sublexical speech sounds (Benson et al., 2001; Hugdahl et al., 2003; Wilson et al., 2004; Rimol et al., 2005; Wilson and Iacoboni, 2006).

An alternative hypothesis that could explain the complexity effect related to the addition of a consonant to form a cluster is that these regions are responding to an increase in phonological working memory due to an increase in sequence length. This is because the addition of a consonant cluster to increase syllabic complexity also increases the length of the sequence. However, previous studies that have manipulated item length to investigate phonological working memory have reported mixed results that seem dependent upon (1) how length was modulated (CV-CCV vs. number of syllables), (2) the type of stimuli used (words, pseudowords), and (3) task demands (passive listening, judgment or naming task) (Okada et al., 2003; Strand et al., 2008; McGettigan et al., 2011). The most consistent finding is that stimulus length defined as the number of syllable yields more reliable results than the addition of consonant clusters. Moreover, if our syllabic manipulation results reflected an increase in phonological working memory, we would expect this contrast to yield clusters of activation within the pre-motor cortex, the IFG, and the IPL, that is, regions that are typically recruited during verbal working memory tasks (Paulesu et al., 1993; Honey et al., 2000; Marvel and Desmond, 2012). However, none of these regions was found in any of our contrasts.

Another alternative hypothesis is that the syllabic effect is due to an increase in acoustic/phonemic complexity. Indeed, consonant clusters are more complex than single consonants both acoustically and phonemically. Given that we parametrically varied both syllabic and supra-syllabic complexity, if this hypothesis were correct, we would expect the same regions to also exhibit an effect of supra-syllabic complexity since the presentation of three different syllables as opposed to the same syllable presented three times also increases acoustical complexity. In addition, we would also expect to see a syllabic complexity ^*^ supra-syllabic complexity interaction driven by a syllabic complexity effect for both simple and complex sequences and a stronger effect of syllabic complexity for the complex sequence. This pattern of result was not found in the SFa or the TTGl or the PTm. However, in the PTm, a region identical to the one reported by Tremblay and Small (2011), sensitivity to the syllabic manipulation was found only for the complex sequences. Combined with the observation that this region is involved in speech production (Dhanjal et al., 2008; Tourville et al., 2008; Peschke et al., 2009; Zheng et al., 2010) and that its activation magnitude varies as a function of syllabic complexity during both speech perception and production (Tremblay and Small, 2011), the result from the current study provides additional support to the hypothesis that the right PT is involved in converting external auditory input into a phonological representation. Our results are in agreement with this hypothesis because an effect of syllabic complexity only emerged in this region when the sequences were composed of three different syllables (i.e., high supra-syllabic complexity). In itself, the addition of a consonant cluster increases the complexity of the syllable template. The additional complexity associated with processing three different sounds (high supra-syllabic complexity) enhances the syllabic manipulation, as three different consonant clusters have to be mapped onto phonological representations as opposed to three single consonants. In sum, the current results lend further support to the notion that regions within the posterior STP are important for the processing of phonological information, perhaps through a template matching mechanisms that uses spectrotemporal information to access stored syllabic representations (Griffiths and Warren, 2002; Warren et al., 2005).

Both the whole-brain and exploratory ROI analyses identified two regions, the STSm and STGa that were sensitive only to the supra-syllabic manipulation. This pattern of response suggests that these regions are involved in tracking changes that affect the structure of the sequence. In the present study, after having heard the second syllable of a sequence, participants could determine whether they would hear the same syllable again (i.e., in the case of simple sequences) or a different syllable (i.e., in the case of complex sequences). Thus, after the second syllable, for simple sequences the continuation was completely deterministic and prediction about upcoming sounds could be made. This pattern of response is also consistent with results from studies that have investigated the perception of speech sounds using a neural adaptation and oddball paradigm. In these studies, cluster of activation were observed within these regions in response to the presentation of a deviant stimulus (Vouloumanos et al., 2001; Joanisse et al., 2007). Overall, the results suggest that these regions are involved in representing sequences overtime. Thus, speech perception mechanisms, even in the absence of a task, are sensitive to changes that affect the structural properties of auditory sequences, consistent with previous work (Tremblay et al., 2012).

Both whole-brain and exploratory ROI analyses also identified a group of regions that was sensitive to both manipulations. These regions included the STGm, the TTGm, the TTSl, the TTSm, the PTa, and the SFp. Sensitivity to both manipulations suggests that these regions do not exhibit a differentiation in processing syllabic or supra-syllabic information. In a previous neuroimaging study using the same parcellation scheme of the STP, both the TTSl and PTa responded to speech and non-speech sounds, whereas the STGm, SFp, and TTGm exhibited an absolute preference for speech sounds (Tremblay et al., 2012), consistent with the idea that regions located anterior and lateral the primary auditory cortex are involved in processing changes in spectro-temporal features (Scott and Johnsrude, 2003). These results suggest that both syllabic and supra-syllabic information recruits common mechanisms involved in processing acoustical information.

In the current study, we explored the neural mechanisms involved in the processing of syllabic and supra-syllabic information during passive speech perception. We demonstrated that both syllabic and supra-syllabic information are processed automatically during passive speech listening, a finding that is consistent with the finding of distinct neural representations for syllable and sequence-level information during speech production (Bohland and Guenther, 2006; Peeva et al., 2010). Importantly, these findings suggest that processing of sublexical information is automatic, at least during the processing of meaningless syllable sequences. Future studies need to examine whether the processing of sub-lexical information is automatic and necessary during language comprehension using more naturalistic stimuli such as words or connected speech. It is possible that the recruitment of phonological mechanisms depends upon the context, or the kind or quality of auditory stimuli being processed. Degraded speech stimuli, for instance, could recruit sublexical phonological mechanisms to a greater extent than high-quality speech sounds. Nevertheless, the present study offers new insight into the functional neuroanatomy of the system involved in sublexical phonological processing, highlighting the importance of the anterior two-thirds of the PT, the primary auditory cortices and the middle part of the STS and STG in these processes.

### Conflict of interest statement

The authors declare that the research was conducted in the absence of any commercial or financial relationships that could be construed as a potential conflict of interest.

## Footnotes

^1^The authors reported several brain areas in which blood-oxygen-level dependent (BOLD) signal magnitude was higher for pseudowords without consonant clusters than for pseudowords containing consonant clusters.

^2^Not all participants took part in all three tasks. Here we report the data from 15 young adults, whereas Bilodeau-Mercure et al. (2014) reported the data for a subset (11) of these participants, who performed the speech perception in noise task.
